# Possible alternative carcinogenesis pathway featuring microsatellite instability in colorectal cancer stroma

**DOI:** 10.1038/sj.bjc.6601141

**Published:** 2003-08-12

**Authors:** N Matsumoto, T Yoshida, K Yamashita, Y Numata, I Okayasu

**Affiliations:** 1 Department of Pathology, Kitasato University School of Medicine, Kitasato 1-15-1, Sagamihara, Kanagawa 228-8555, Japan; 2 Department of Pathology, Kitasato University East Hospital, Asamizodai, Sagamihara, Kanagawa 228-8520, Japan

**Keywords:** microsatellite instability, stroma, epithelium, colorectal cancer, p53

## Abstract

Differential microsatellite instability (MSI) in tumour epithelial and stromal compartments has not been well examined for colorectal cancers. Using laser-captured microdissection, separate specimens of these compartments of 40 sporadic colorectal cancers were sampled and MSI was tested with four markers. To examine the relation between the MSI phenotype in the stroma and other genetic events and histopathological features, p53 and K-*ras* gene mutations were analysed, and the expression of p53, hMLH1, and hMSH2 protein was determined by immunohistochemistry. Microsatellite instability positive results were obtained for both epithelium (34%) and stromal tissue (41%). While MSI in epithelium correlated with differentiation and Dukes' stage, that in stroma demonstrated an inverse relation, being particularly frequent in well-differentiated adenocarcinomas (54%) and Dukes' A lesions (55%). Further, a significant inverse correlation between p53 protein overexpression in the epithelium and MSI in the stroma was found (*P*=0.02475). The results suggest an alternative pathway of carcinogenesis involving stromal genetic instability in the development of colorectal cancers.

Interactions between epithelial and mesenchymal cells in the various organs play important roles in their development (Marsh and Trier, 1974; Shekhar *et al*, 2001), differentiation (Camps *et al*, 1990; Hom *et al*, 1998) and growth (Camps *et al*, 1990; Hom *et al*, 1998). However, the contribution of stromal cells to the generation and progression of epithelial neoplasia has not been thoroughly investigated. Although genetic alterations are frequently observed in many benign and malignant epithelial tumours in the form of microsatellite instability (MSI) (Michael-Robinson *et al*, 2001; Ward *et al*, 2001), the possibility of genetic abnormalities in the background microenvironment of these tumours has generally not been addressed properly. However, recent findings indicate that loss of heterozygosity (LOH) may be frequent in mammary stromal tissue in breast cancer patients (Moinfar *et al*, 2000). In one series of sporadic colorectal cancers, it was found that approximately 15% of tumours demonstrated MSI (Michael-Robinson *et al*, 2001). Since data on MSI in stroma of colorectum have hitherto not been published, to our knowledge, here we investigated this point with reference to clinicopathological features of colorectal cancers, using laser-captured microdissection. A recent study showed high p53 gene mutation loads in ulcerative colitis with an inflammatory microenvironment predisposed to colorectal carcinoma (Hussain *et al*, 2000; Yoshida *et al*, 2003). To test the hypothesis that genetic instability in the p53 locus stage might be a key early event in tumorigenesis, with change in the stroma possible influencing epithelial tumorigenesis, microsatellite markers D17S796 (Gyapay *et al*, 1994), TP53 and D17S786 (Gyapay *et al*, 1994), located in the short arm of chromosome 17 within 4 cM proximity to the p53 gene (17p13), and D17S579 (17q21) (Anderson *et al*, 1993) in the long arm of the chromosome 17 were analysed here in a series of sporadic colorectal cancers.

## MATERIALS AND METHODS

### Samples and preparation

In total, 40 surgically resected sporadic colorectal adenocarcinomas from patients, 24–89-years old, undergoing treatment at Kitasato University Hospital and Kitasato University East Hospital, were randomly selected. Histological typing was performed according to the criteria of the Japanese Society for Cancer of the Colon and Rectum (Jinnai, 1983) and also Dukes' classification.

**Figure 1:**
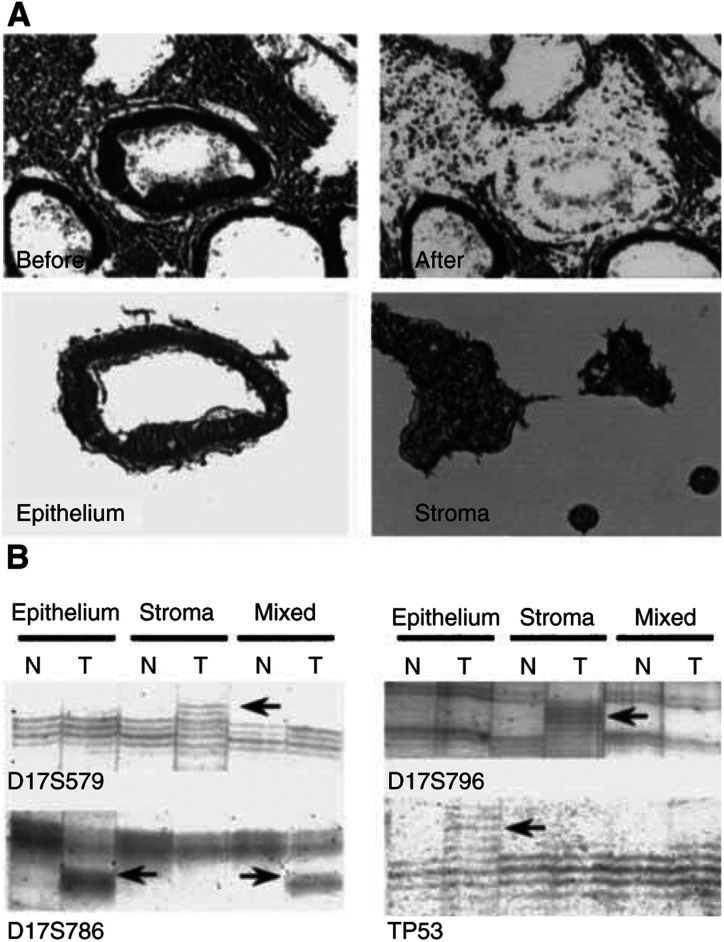
(**A**) Example of microdissection of epithelial cells and adjacent stromal cells from a single histologic section. Original magnification, × 100, (**B**) Representive data from microsatellite analysis of sporadic colorectal cancers. Arrows indicate shifted bands reflecting microsatellite instability. N=normal mucosa; T=tumour tissue.

On surgical removal, tissues were immediately frozen with liquid nitrogen in OCT compound for storage at −80°C. Frozen sections 10 (10 *μ*m thick) were fixed in 70% alcohol and stained with Mayer's haematoxylin. Neoplastic epithelial and adjacent stromal tissues in the lamina propria were carefully microdissected with a laser-captured microdissection system (LM200, Arcturus, Mountain View, CA, USA) to avoid contamination (Figure 1A

). Tissues from whole sections, including both epithelial and stromal elements (mixed), were also manually dissected. Tumour tissues with strong inflammatory cell infiltration were excluded from study. Likewise, normal mucosa from the colorectum of the same patients was sampled and processed as an internal control. Tissues were lysed in sodium dodecyl sulphate-lysis buffer with proteinase K, and DNA was extracted with the standard phenol–chloroform–ethanol precipitation method.

### Microsatellite analysis

The polymerase chain reaction (PCR) was performed for four microsatellite markers, D17S796, TP53 (forward: 5′-ACTGCCACTCCTTGCCCCATTC-3′, reverse: 5′-CACCTCGGGCTGAATAGTATCCCT-3′), D17S786, and D17S579, selected for analysing allelic instability in chromosome 17. Microdissected DNA (5–10 ng) was amplified with a Rapid Cycler (Idaho Technology, Idaho falls, ID, USA) and *Takara Ex Taq* DNA polymerase (Takara, Kyoto, Japan) under conditions as follows: 94°C for 3 min as the initial step, 35 cycles of 0 s at 94°C, 0 s at an appropriate temperature for each marker amplification, 6 s at 74°C, and a final step of 74°C for 3 min. The PCR products were fractionated by 3.6% polyacrylamide gel electrophoresis, fixed with 10% formamide and visualised using a Silver Stain Plus Kit (Bio-Rad, Hercules, CA, USA).

Results for normal tissues were considered informative when two or three (because some samples were insufficient for PCR amplification even if the experiment was repeated) of three samples, including epithelium, stroma, and mixed tissue DNA from each case, exhibited identical banding patterns. In pairs with informative normal tissue, sufficient PCR amplification of tumour tissues was considered as informative and MSI was defined as either a marked alteration in repeat length or as a new discrete band above or below the expected allele (Figure 1B). We referred to a previous report to judge whether the samples were informative (Luttges *et al*, 2001). Cases for which pairs of samples were informative for two of four markers were defined as informative and those showing MSI positivity (MSI+) for at least one marker were considered as MSI+.

### Immunohistochemistry and p53 and K-*ras* gene analysis

To analyse p53, hMLH1, and hMSH2 protein expression and for assessment of p53 and K-*ras* gene mutations, tissues were fixed routinely in 10% buffered formalin and embedded in paraffin. Serial sections (3 *μ*m thick) were applied for haematoxylin and eosin staining, immunohistochemistry, and mutation analyses.

**Figure 3:**
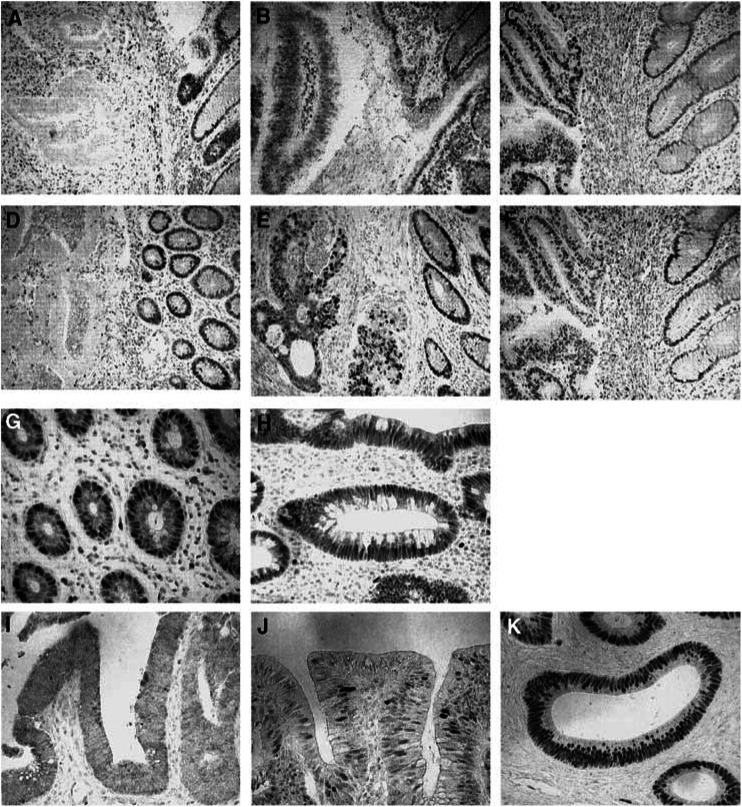
Expression of hMLH1, hMSH2, and p53 protein. Examples of hMLH1 (**A–C**) and hMSH2 (**D–F**) protein immunostaining in tumorous epithelial cells. (**A**) and (**D**) − in epithelial cells; + in stromal cells (**B**) and (**E**) + in epithelial cells; + in stromal cells. (**C**) and (**F**) ++ in epithelial cells; ++ in stromal cells. Original magnification, × 200. (**G**) hMLH1 protein expression in normal stromal cells. (**H**) Loss of hMLH1 protein expression in tumorous stromal cells from the same case. Original magnfication, × 400. Examples of p53 protein immunostaining in sporadic colorectal cancers (**I–K**). (**I**) − for p53 protein expression; (**J**) +; (**K**) ++. Original magnification, × 400.

Immunohistochemical staining was performed with monoclonal anti-p53 (DO7, × 300 dilution, Novocastras Lab., Newcastle, UK), monoclonal anti-hMLH1 (Clone; G168-15, × 200 dilution, BD PharMingen, San Diego, CA, USA) and monoclonal anti-hMSH2 (Clone; G219-1129, × 500 dilution, BD PharMingen) antibodies, using the standard labelled streptavidin–biotin–peroxidase complex method described in our previous report (Yamashita *et al*, 2001). The amounts of positive cells were expressed as the percentage of the total number of epithelial cells and assigned to one of three categories for p53 : ++, > 50%; +, 0.5–50%; −, < 0.5% (Figure 3I–K

), for hMLH1 and hMSH2 : ++, > 50%; +, 10–50%; −, < 10% (Figure 3A–F). We also examined the expression of hMLH1 and hMSH2 proteins in stromal cells, with classification into : ++, strong staining; +, focal staining; −, negative staining. Definite nuclear staining of adjacent non-neoplastic epithelial and stroma cells or lymphocytes served as internal positive controls.

p53 and K-*ras* gene mutations were analysed by the PCR-single strand conformation polymorphism (SSCP) method described in our previous report (Yamashita *et al*, 2001). Mutations were detected as abnormally shifted bands.

### Statistics

Differences for each category of clinicopathological features, with reference to MSI in the epithelium and stroma were examined using the Fisher' s exact and *χ*^2^ tests.

## RESULTS

### MSI status

Microsatellite instability was frequently detected in epithelial and stromal areas of sporadic colorectal cancers (Table 1

**Table 1:** MSI frequencies for each microsatellite marker

	**MSI+ samples/informative samples**			**MSI+ samples/informative samples**
**Marker**	**Epithelium**	**Stroma**	**P-value**	**Mixed tissue**
D17S796	4/36 (11%)	8/33 (24%)	0.1506	1/38 (3%)
TP 53	6/35 (17%)	6/35 (17%)	0.9999	5/37 (14%)
D17S786	6/37 (16%)	1/36 (3%)	0.0512	3/40 (8%)
D17S579	4/36 (11%)	3/37 (8%)	0.6631	4/39 (10%)
				
Total	13/38 (34%)	16/39 (41%)	0.5372	9/40 (23%)

Significance was determined by the *χ*^2^ test (epithelium *vs* stroma). Total refers to informative cases that showed MSI+ for at least one marker.

). Of the 40 colorectal cancers studied, 13 out of 38 informative cases (34%) were MSI+ for one or more of the markers in tumour epithelium, 16 out of 39 cases (41%) in adjacent stromal areas, and nine of 40 (23%) in mixed tissue. MSI+ for two or more markers was found in three cases in the epithelium and two in stroma. While MSI was slightly more common in the stroma than epithelium, the difference did not reach significance (Table 1). For each component, only three cases had MSI in both epithelium and stroma for the same markers (two for TP53 and one for D17S796), suggesting appropriate microdissection without contamination. Others showed MSI specific to the epithelium alone, stroma alone, epithelium and mixed tissue (Figure 1B; D17S786), or stroma and mixed tissue. All the tumours with MSI in mixed tissue also demonstrated MSI in epithelium and stroma. A comparison of MSI frequency for each marker between the epithelium and stroma revealed stromal MSI+ to be less frequent (1/36=3%) than epithelial MSI+ (6/37=16%) for D17S786 (*P*=0.0512) (Table 1). With D17S796, MSI was more often found in the stroma (8/33=24%) than in the epithelium (4/36=11%), without significance (Table 1). No differences were found for D17S579 and TP53. Concerning the low MSI frequency for mixed tissue as compared with the epithelium or stroma alone, MSI−DNA might disturb the positivity with MSI-PCR due to lowered sensitivity.

### Histopathological features of epithelial and stromal MSI status

Histopathological and molecular features of the sporadic colorectal cancers are detailed in Table 2

**Table 2:** MSI findings for the epithelium and stroma, with reference to tumour features

		**MSI+ cases /informative cases**			**MSI+ cases/informative cases**
**Variable**	**Total cases**	**Epithelium**	**Stroma**	**P-value**	**Mixed tissue**
*Location*					
Right side	14	6/14 (43%)	7/14 (50%)	0.3549	4/14 (29%)
Left side	26	7/24 (29%)	9/25 (36%)	0.1388	5/26 (19%)
*Differentiation*					
Well	14	2/13 (15%)	7/13 (54%)	0.0393*	2/14 (14%)
Moderate	16	6/15 (40%)	8/16 (50%)	0.5761	5/16 (31%)
Poor	10	5/10 (50%)	1/10 (10%)	0.0510**	2/10 (20%)
*Dukes' stage*					
A	11	0/10 (0%)	6/11 (55%)	0.0057*	1/11 (9%)
B	12	6/12 (50%)	5/12 (42%)	0.6820	4/12 (33%)
C	17	7/16 (44%)	5/16 (31%)	0.4652	4/17 (24%)
*p53 mutation*					
+	15	4/13 (31%)	5/14 (36%)	0.7854	4/15 (27%)
−	25	9/25 (36%)	11/25 (44%)	0.5637	5/25 (20%)
*K-ras mutation*					
+	12	5/11 (45%)	6/11 (55%)	0.6698	4/12 (33%)
−	28	8/27 (30%)	10/27 (37%)	0.6307	5/28 (18%)
*p53 protein*					
−	7	1/7 (14%)	3/7 (43%)	0.5594	1/7 (14%)
+	10	3/10 (30%)	7/10 (70%)	0.0943**	3/10 (30%)
++	23	9/21 (43%)	6/22 (27%)	0.5127	5/23 (22%)
*hMLH1 protein*					
−		2/4 (50%)^a^	1/1 (100%)^b^		
+		0/6 (0%)	12/30 (40%)		
++		11/27 (40%)	3/7 (43%)		
*hMSH2 protein*					
−		0/1 (0%)^a^	0/0 (0%)^b^		
+		3/9 (33%)	16/35 (46%)		
++		10/25 (40%)	0/2 (50%)		

Significance was determined by the *χ*^2^ test and Fisher's exact test (epithelium *vs* stroma).‘MSI+ cases’ refer to cases that showed MSI+ for at least one marker.

* *P* < 0.05

** *P* < 0.1

a *n* = The cases of hMLH1 or hMSH2 protein expression in epithelial cells.

b *n* = The cases of hMLH1 or hMSH2 protein expression in stromal cells.

**Figure 2:**
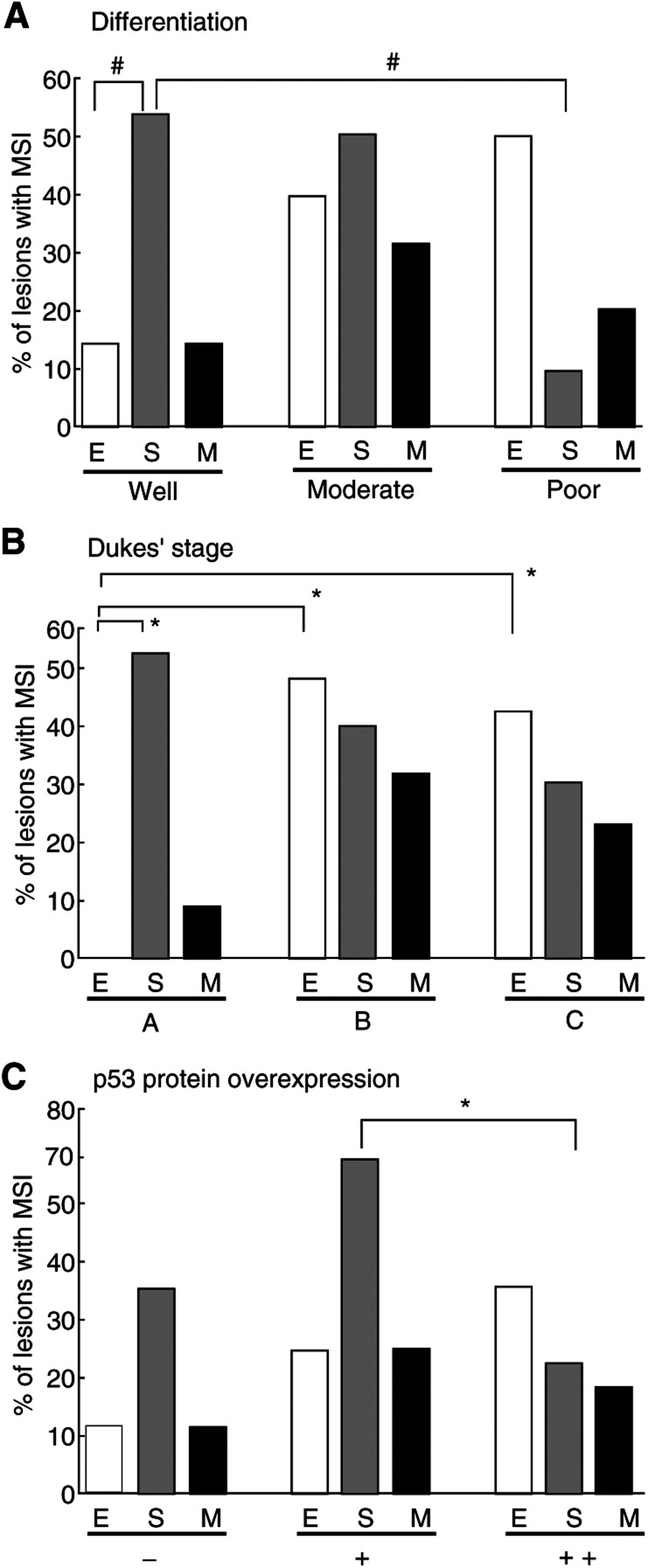
Data for MSI with reference to tumour features. E=epithelium; S=stroma; M=mixed tissue. ^*^P<0.01; ^#^*P*<0.05.

. MSI frequencies differed between epithelium and stroma in well-differentiated (*P-*value=0.0393) and poorly differentiated (*P-*value=0.0510) (Figure 2A

) adenocarcinomas. Stromal MSI+ was more often detected in well-differentiated adenocarcinomas (7/13=54%) than in poorly differentiated cancers (1/10=10%) (*P-*value=0.0286), whereas the frequency of epithelial MSI+ correlated with progression (Figure 2A). With analysis of Dukes' stage, although there was a tendency for an inverse relation with stromal MSI+ (Figure 2B), it did not reach significance (*P-*value=0.4807). In contrast, epithelial MSI+ showed significant variation with the Dukes' stage (*P-*value=0.0277) (Figure 2B). A significant correlation was also detected between epithelial MSI+ and stromal MSI+ and Dukes' stage (*P-*value=0.0455). It is notable that in stage A lesions, stromal MSI+ (6/11=55%) was more frequent than epithelial MSI+ (0/10=0%) (*P-*value=0.0057) (Figure 2B).

### MSI status, p53 gene mutations, and p53 protein overexpression

A significant inverse correlation between p53 protein overexpression in epithelium and MSI in stroma was found (7/10=70% in ‘+’ cases *vs* 6/22=27% in ‘++’ cases) (*P-*value=0.02475) (Figure 2C). p53 gene mutations were detected in 15 out of 40 (38%) tumours with no significant differences observed with reference to epithelial and stromal MSI (Table 2).

### MSI status and K-*ras* gene mutation

K-*ras* gene mutations were detected in 12 out of 40 (30%) tumours with no significant differences observed with reference to epithelial and stromal MSI (Table 2).

### hMLH1 and hMSH2 protein expression in epithelial and stromal MSI

Losses of hMLH1 and hMSH2 expression were detected in 10% (4/40) and 3% (1/40) of cases in the epithelium and 3% (1/40) and 0% (0/40) in the stroma, respectively. No significant correlations between hMLH1 and hMSH2 protein expression in epithelial or stromal cells and epithelial MSI+ or stromal MSI+ were found (Table 2) (*P-*value not shown).

## DISCUSSION

Regarding interactions between the epithelium and stroma, several hypotheses have been proposed to explain fibroblast-promoting effects on tumour growth. Most of the intercellular material, the extracellular matrix (ECM) molecules that are required for tumour growth and progression, is produced by stromal cells (Noel *et al*, 1998). It has been demonstrated that neoplastic breast stroma drives alteration in gene expression as compared with normal tissue (Leygue *et al*, 1998). In fact, it is generally believed that the epithelium is the neoplastic element in most tumours and that altered gene expression in stroma occurs as the secondary reaction. However, the recent finding of frequent genetic changes in mammary stromal tissue in breast cancer patients (Moinfar *et al*, 2000), and the demonstration that inflammation-associated stroma promotes conversion of colonic adenoma cells to adenocarcinoma cells in nude mice (Okada *et al*, 2000) suggest a more complex scenario.

Our present study showed that MSI in stromal and epithelial elements can occur independently in sporadic colorectal cancers, in line with the previous findings for breast carcinomas (Kurose *et al*, 2001). Further, while MSI in the epithelium tended to correlate with differentiation and the Dukes' stage, the inverse was the case for MSI in stroma. These interesting results strongly suggest that there are alternative mechanisms involving stromal MSI operating in colorectal carcinogenesis and progression. According to Young *et al*, methylation of CpG island occurs both in the epithelium and stroma (Young *et al*, 2001). Stromal MSI presented in this study might be due to the methylation of mismatch repair enzymes in stromal cells, although the identification of the enzyme remains unclear. Previously, it was shown that high-level MSI (MSI-H) tumours are more likely to be right sided than their low-level MSI (MSI-L) or MSI stable counterparts (Michael-Robinson *et al*, 2001; Ward *et al*, 2001). In the present study, while MSI frequencies in both the epithelium and stroma were high in right-side (43, 50%) as compared to left-side lesions (29, 36%), the difference did not reach statistical significance. This might be due to relatively small numbers of examined cases or inclusion of both MSI-L and MSI-H results in our analysis. Additionally, we examined the expression of DNA mismatch repair enzymes, hMLH1 and hMSH2 protein, in epithelial and stromal cells, respectively, as the MSI-H phenotype has been suggested to be of importance for the DNA mismatch repair system in sporadic colorectal cancers (Dietmaier *et al*, 1997; Ward *et al*, 2001). In the present study, epithelial MSI+ for two or more markers was found in three cases, two of which showed loss of hMLH1 protein expression. In stroma, MSI+ for two or more markers was found in two cases, one of which showed loss of MLH1 in stromal cells (Figure 3G and H). Although we have not used the standard markers that were recommended for MSI analysis on the basis of a National Cancer Institute Workshop (Boland *et al*, 1998), the results of hMLH1 and hMSH2 protein expression provide support for the validity of our study of MSI in epithelial and stromal cells. NCI-recommended standard markers are two mononucleotide repeat markers and three dinucleotide markers, but all of the markers we used in this study are dinucleotide repeat markers ((CA)_*n*_ repeat). Therefore, it is difficult to compare MSI-H entity with our MSI+ for two or more markers. Additionally, discordance of the loss of hMLH1 or hMSH2 and our MSI+ tumours could occur in the present study.

Although no significant links were found between MSI and p53 or K-*ras* gene mutations, MSI in the epithelium, but not stroma, tended to correlate positively with p53 protein overexpression. Recently, Kapiteijn *et al*. (2001) reported that p53 gene mutation corresponds more often to p53 overexpression in left- than in right-sided tumours, suggesting that mechanisms of oncogenesis may differ between the two cases. This is in line with our results for a greater prevalence of p53 overexpression with p53 gene mutations in left-sided tumours (9/11, 81%), than in those found on the right side (1/4, 25%) (*P*=0.0390, data not shown). Furthermore, we found a difference in the relation between p53 mutations and stromal MSI (stromal MSI+/p53 mutation, 3/11 (27%) in the left-side; 3/4 (75%) in the right-side, *P*=0.0952). These results support the conclusion of Kapiteijn *et al*. (2001) and suggest a relation of p53 mutation to stromal MSI+.

From the available data, contrary to the general belief that abnormalities in stroma occur as reactions to epithelial tumour cells, we propose the hypothesis that an alternative pathway may exist, with stromal genetic instability influencing epithelial cells in carcinogenesis.
